# A Multi-Level Fit-Based Quality Improvement Initiative to Improve Colorectal Cancer Screening in a Managed Care Population

**DOI:** 10.1038/s41424-018-0046-z

**Published:** 2018-09-04

**Authors:** Christine Yu, Samuel Skootsky, Mark Grossman, Omai B. Garner, Anna Betlachin, Eric Esrailian, Daniel W. Hommes, Folasade P. May

**Affiliations:** 1 The Vatche & Tamar Manoukian Division of Digestive Diseases, Department of Medicine, David Geffen School of Medicine, University of California, Los Angeles, CA USA; 2 Department of Medicine, David Geffen School of Medicine, University of California, Los Angeles, CA USA; 3 Department of Pathology and Laboratory Medicine, David Geffen School of Medicine, University of California, Los Angeles, CA USA; 4 Veterans Affairs Greater Los Angeles Healthcare System, Los Angeles, CA USA; 5 VA HSR&D Center for the Study of Healthcare Innovation, Implementation and Policy (CSHIIP), Los Angeles, CA USA; 6 UCLA Kaiser Permanente Center for Health Equity, Los Angeles, CA USA

## Abstract

**Introduction:**

Colorectal cancer (CRC) is a common but largely preventable disease with suboptimal screening rates despite national guidelines to screen individuals age 50–75. Single-component interventions aimed to improve screening uptake only modestly improve rates; data suggest that multi-modal approaches may be more effective.

**Methods:**

We designed, implemented, and evaluated the impact of a multi-modal intervention on CRC screening uptake among unscreened patients in a large managed care population. Patient-level components included a mailed letter with education about screening options and pre-colonoscopy telephone counseling. For providers, we facilitated communication of screening test results and work-flow for abnormal results. System-level modifications included establishment of a patient navigator, expedited work-up for abnormal results, and stream-lined colonoscopy scheduling. We measured the rate of screening uptake overall, screening uptake by modality, change in the proportion of the population screened, and positive fecal immunochemical test (FIT) follow-up rates in the 1-year study period.

**Results:**

There were 5093 patients in the intervention cohort. Of these, 33.2% participated in FIT or colonoscopy screening within 1 year of the mailing. A total of 1078 (21.2%) participants completed a FIT and 611 (12.0%) completed a screening colonoscopy. The screening rate in the managed care population increased from 65.1 to 76.6%. Fifty-nine patients (5.5%) had a positive FIT, of which 30 (50.8%) completed a diagnostic colonoscopy.

**Conclusion:**

Multi-modal interventions can result in substantial improvement in CRC screening uptake in large and diverse managed care populations.

**Translational Impact:**

Health systems should shift their focus from single-level to multi-level interventions when addressing barriers to CRC screening.

## Introduction

In the United States, colorectal cancer (CRC) is the third most commonly diagnosed cancer and the second most common cause of cancer-related mortality in men and women^1–4^. Incidence and mortality have declined over the past 2 decades, largely due to substantial national public health and screening efforts^1,2^. However, screening uptake remains subpar. Currently, only 65% of eligible adults aged 50–75 years are screened^5^, far below the National Colorectal Cancer Roundtable (NCCRT) goal to screen 80% of Americans by 2018.

The United States Preventive Services Task Force (USPSTF) reaffirmed the importance of screening average-risk Americans aged 50 to 75 in their 2016 updated CRC screening guidelines^6^. Given the lack of comparative effectiveness studies demonstrating superiority of any single screening strategy, the USPSTF highlighted the importance of maximizing adherence to screening and implied a strong role for patient preference in screening method utilization. More recently, the Multi-Society Task Force (MSTF) guidelines recommended fecal immunochemical test (FIT) and colonoscopy as first-tier screening tests, again emphasizing the importance of offering FIT in screening programs^7^. Overall, these guidelines are encouraging for health systems that offer low-cost and convenient modalities like FIT in addition to colonoscopy for screening^8^.

Achieving a high CRC screening rate can be challenging in large health systems and health maintenance organizations (HMOs) with diverse patient populations and large provider groups. Screening rates are as low as 26% in such settings and rarely reach rates over 69% without significant system supports^9–11^. Single-component interventions aimed to improve CRC screening uptake in integrated health systems have included changes to health plan policies and practices^12^, enhanced patient-test interaction^13–16^, FIT mailings programs^10,17,18^, reminder systems^19^, financial incentives^20^, and use of patient navigators^11^. While many of these efforts have shown some success in augmenting screening rates, none have raised rates over 80%, and most leave multiple mutable barriers to screening unaddressed.

Multi-modal interventions aimed to increase uptake of CRC screening via integrated patient-, provider-, and system-level components may be more effective; however, prospective data examining their impact in managed care settings is limited^13,21^. Florea et al.^22^ evaluated a multi-modal intervention to increase CRC screening uptake in three federally qualified health centers serving a predominantly low income and underinsured population. Their intervention incorporating staff and provider education, patient decision aids and an electronic medical record (EMR)-based reminder system led to 6.3–6.9% increases in CRC screening uptake. Faced with suboptimal CRC screening rates among managed care patients in our academic health center, we began a multi-disciplinary quality improvement (QI) initiative. Led by leadership in both internal medicine and gastroenterology (GI), our initiative aimed to develop and evaluate the impact of a multi-component intervention on CRC screening rates in our population of unscreened managed care patients.

## Methods

### Setting and population

In 2014, the UCLA Medical Group, a risk-bearing organizational unit of UCLA Health, collaborated with the UCLA GI QI team to design a CRC screening initiative for the managed care population. UCLA Health is a large academic medical center with defined primary care populations and robust referral-based care. The primary care population is defined by patients enrolled in risk-based commercial HMO contracts that receive coverage for care services only via UCLA Medical Group as part of the California delegated model. The population includes 60,000 patients with a baseline CRC screening rate of 65.1% in those eligible for CRC screening. The aim of this QI was to increase CRC screening uptake in this patient population.

With assistance from a data analyst from the UCLA Value Analytics (VA) team, we used EMR, billings data, and claims data to identify the cohort of managed care patients due for CRC screening. We targeted patients within the managed care population aged 51 to 75 who were not up-to-date with screening, defined by a lack of FIT or fecal occult blood testing (FOBT) in the past year, flexible sigmoidoscopy in the past 5 years, or colonoscopy in the past 10 years, consistent with NCQA Healthcare Effectiveness Data and Information Set (HEDIS) specifications^23^. Patients also required a valid mailing address to be included. Exclusion criteria included a personal history of CRC and prior total colectomy.

### Overview of intervention and implementation

We evaluated the impact of the multi-component intervention on CRC screening rates. The intervention included patient-level, provider-level, and system-level features. All components were initiated simultaneously and were based on programs previously demonstrated to increase CRC screening rates in similar settings^10,11,17,18^ or tailored to address specific barriers at our institution (Table 1). We then evaluated the screening uptake by FIT and by colonoscopy after QI implementation. The UCLA institutional review board deemed our work exempt systems improvement consistent with ongoing hospital QI efforts.

**Table 1:** Intervention components to improve colorectal cancer (CRC) screening uptake

Patient-level components	Provider-level components	System-level components
Mailed CRC education and FIT kit	Notification of QI goals	Stream-lined work-flow for abnormal screening test results
Introduction of patient navigator with interactive telephone counseling	Enhanced communication of screening results	Improved process for colonoscopy scheduling
	Improved work-flow for abnormal FIT results	

#### Patient-level interventions

Each eligible patient received one or two mailed letters between June 2015 and October 2015 with an enclosed FIT kit. The first wave of letters was sent to patients who met eligibility criteria on 16 June, 2015. A second wave of identical letters was sent to patients that had not completed screening in the 4-month time interval after the initial mailing and to a small group of managed care patients newly eligible for screening on 16 October, 2015. The letter informed patients of the national CRC screening recommendation and described both FIT and colonoscopy screening. FIT kits were OC-Auto FIT-CHEK kits made by Polymedco, Inc. and contained a self-addressed mailer and postage for the UCLA laboratory. Patients were asked in the letter to either complete the FIT kit or to call and schedule an appointment with their primary care provider (PCP) to discuss colonoscopy and alternative forms of screening. Patients were also provided a form to submit information about screening performed outside UCLA Health.

Patients with positive FITs were informed by their PCPs. A patient navigator then worked alongside GI schedulers to contact the patient to schedule the colonoscopy and provide peri-colonoscopy instructions. Our patient navigator was a registered nurse with interactive telephone counseling training on how to provide diet, bowel preparation, and medication management for colonoscopy. The patient navigator also served as a liaison between schedulers and patients when scheduling screening colonoscopies through the open-access endoscopy facility.

#### Provider-level interventions

Provider-level components included education about the CRC screening program and improved communication between GI and primary care teams regarding positive FIT results. We informed PCPs about the planned QI to increase CRC screening rates through email notifications. When a FIT was negative, the patient’s PCP received an EMR inbox message to review results and enter updated screening data into the Health Maintenance (HM) tab. The HM tab is an electronic portal within each patient’s EMR chart where providers can view, enter, and reference the status of preventive measures like cancer screening for individual patients.

For all positive FIT results, PCPs received a personalized email generated by a member of the QI team informing them of the positive result and offering assistance with coordination of a diagnostic colonoscopy. PCPs were asked to verify that colonoscopy was appropriate and, if so, to place an electronic request for one. Our patient navigator then contacted the patient to schedule the procedure as described above. Endoscopists completed diagnostic colonoscopies, reviewed pathology results to determine surveillance colonoscopy intervals, communicated results to patients via phone or mailed letters and entered colonoscopy results and surveillance recommendations into the HM tab. For patients with findings concerning for malignancy, endoscopists ordered appropriate cross-sectional imaging and referrals to colorectal surgery and oncology.

#### System-level interventions

System-level interventions included coordination of care processes among our QI team, PCPs, the GI division, and the scheduling department to achieve diagnostic colonoscopies for FIT-positive patients. Once the PCP placed an electronic request for a diagnostic colonoscopy, the procedure was scheduled by GI scheduling. If the patient could not be reached, the scheduler left a voice message prompting patients to call back. If a call back was not received, additional efforts included a second phone call and an electronic message to the PCP to re-refer the patient for colonoscopy when appropriate. Diagnostic colonoscopies were prioritized for completion within 6 months. The GI QI team verified all screening results and surveillance intervals and entered any missing data.

### Statistical analysis

Our primary outcomes of interest were the rate of screening uptake after the intervention and the change in the proportion of the managed care population screened after the intervention. For all analyses, we excluded patients who reported up-to-date screening. Screening uptake was measured at 30-day intervals and at 1 year after receipt of the first mailed FIT kit. We calculated the pre-intervention screening rate as the percentage of screening-eligible patients in the managed care population that were up-to-date with screening on 16 June, 2015 where the denominator was the total number of managed care patients eligible for CRC screening, and the numerator was the number of these patients that met HEDIS screening requirements. Post-intervention screening rates were calculated at 30, 60, 90, 180, 270, and 365 days after the start of the intervention.

Additionally, we examined screening uptake and method of screening used by age, sex, race, and ethnicity. We used one mutually-exclusive variable that combined concepts of race and ethnicity by including Hispanic as a primary race (non-Hispanic white, non-Hispanic black, Hispanic, Asian, other). For patients with a positive FIT, we examined the rate of and time to diagnostic colonoscopy. Finally, we investigated screening and diagnostic colonoscopy findings.

## Results

### Descriptive characteristics and screening uptake

**Fig. 1:**
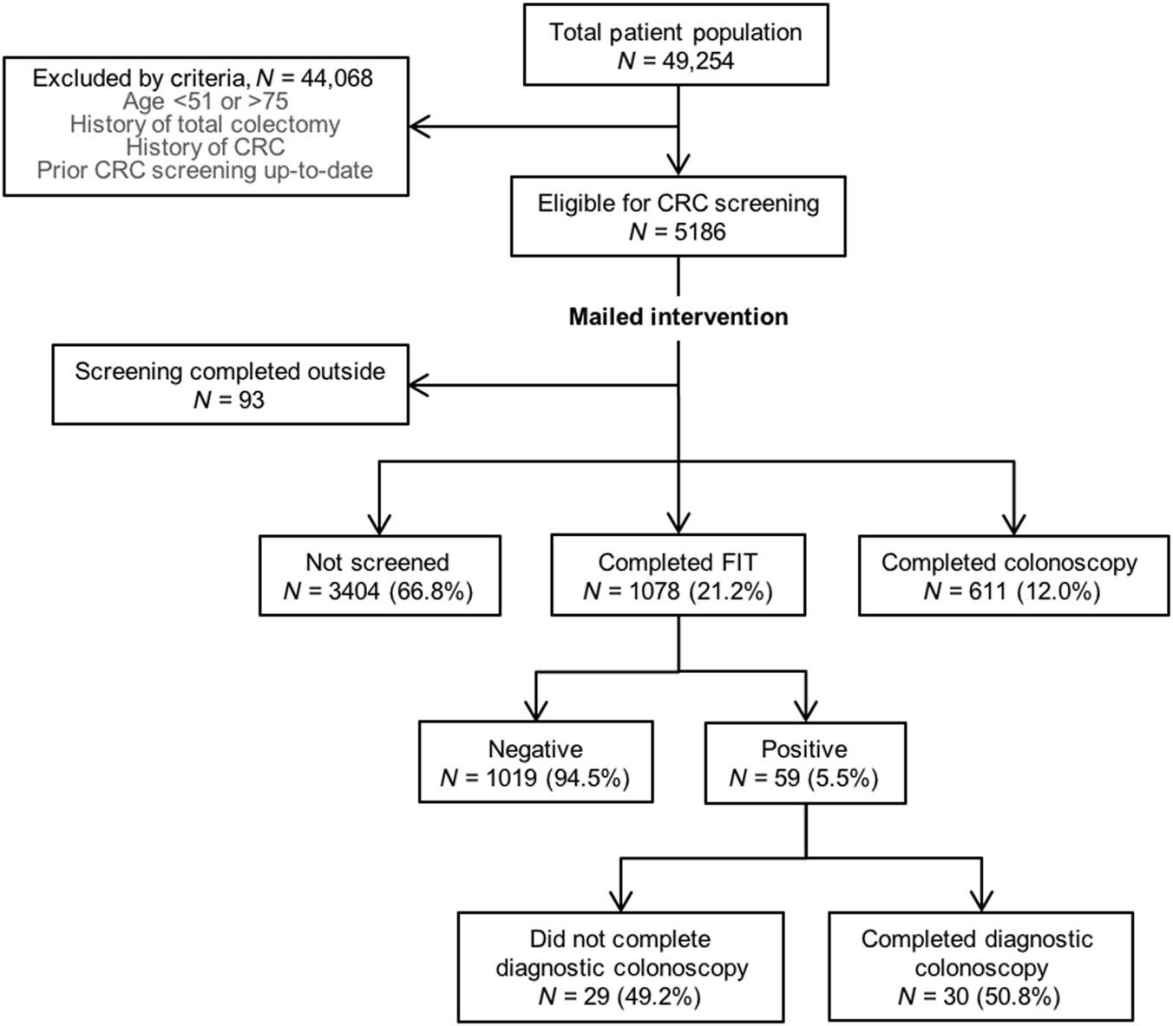
Intervention cohort by study inclusion and exclusion criteria

Intervention letters were mailed to 5186 patients. There were 93 patients that reported up-to-date screening, leaving 5093 patients in the final intervention cohort (Fig. 1). The cohort was 55.5% female, 50.3% white, 7.9% black, 12.4% Hispanic, 10.1% Asian, and 5.8% other race/ethnicity. The mean age was 61.1 years (Table 2).

**Table 2:** Demographic characteristics of the intervention cohort and overall CRC screening uptake at 1 year, *N* = 5093

	Total *N* (%) or mean (s.d.)	*N* (%) screened (FIT or colonoscopy)	*N* (%) screened by FIT	*N* (%) screened by colonoscopy
Age	61.1 ( ± 7.5)	62.1 ( ± 7.6)	63.1 ( ± 7.5)	60.2 ( ± 7.3)
Sex				
Male	2266 (44.5)	712 (31.4)	442 (62.1)	270 (37.9)
Female	2827 (55.5)	977 (34.6)^a^	636 (65.1)	341 (34.9)
Race/ethnicity				
White	2562 (50.3)	866 (33.8)	548 (63.3)	318 (36.7)
Black	403 (7.9)	142 (35.2)	84 (59.2)	58 (40.8)
Hispanic	632 (12.4)	228 (36.1)	143 (62.7)	85 (37.3)
Asian	515 (10.1)	235 (45.6)^b^	145 (61.7)	90 (38.3)
Other	296 (5.8)	103 (34.8)	69 (67.0)	34 (33.0)
Unknown	685 (13.5)	115 (16.8)^b^	89 (77.4)^b^	26 (22.6)^b^
Total	5093	1689 (33.2)	1078 (63.8)	611 (36.2)

*CRC* colorectal cancer, *FIT* fecal immunochemical test, *s.d.* standard deviation

^a^Signifies significant difference in screening rate between males and females at *p* < 0.05

^b^Signifies significant difference in screening rate from whites at *p* < 0.05

**Fig. 2:**
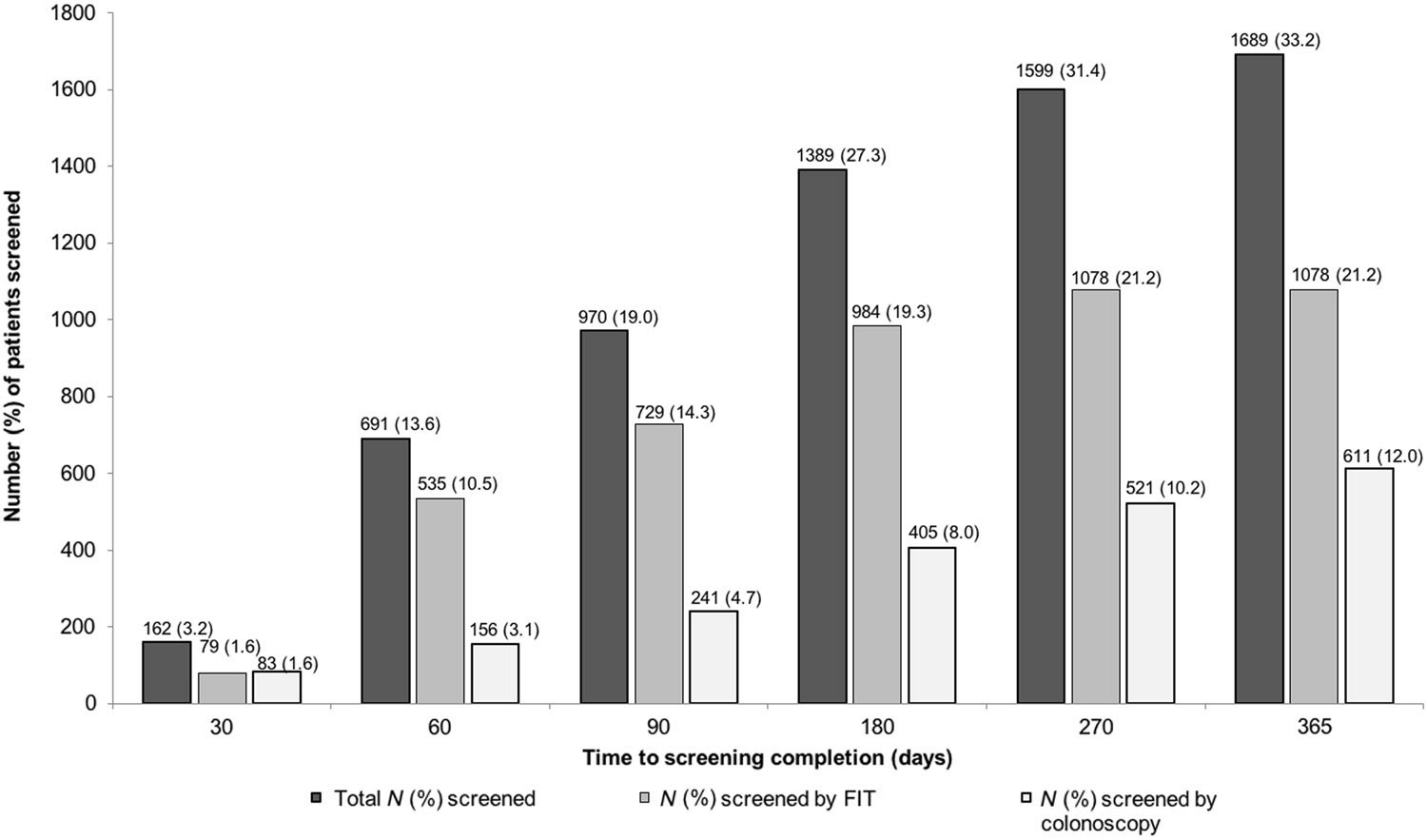
Colorectal cancer (CRC) screening uptake within intervention cohort at 30 days, 60 days, 90 days, 180 days, 270 days, and 365 days; *N* (%)

Overall, 33.2% of patients participated in FIT (21.2%) or colonoscopy (12.0%) screening within 1 year of the mailed letter screening. The CRC screening rate for the screen-eligible managed care population was 65.1% prior to the start of the intervention and increased to 76.6% after the 1-year study period. Screening rates at 30-day intervals are provided in Fig. 2. The percent of patients screened increased for each 30-day interval from 3.2% at 30 days to 33.2% at 1 year. Screening uptake was higher in women than in men (34.6% vs. 31.4%, *p* = 0.02) and higher in Asians than in whites (45.6% vs. 33.8%, *p* < 0.001).

### Screening uptake by modality and screening colonoscopy findings

FIT was the most common screening modality (1078, 63.8%). Of those screened, 611 (36.2%) patients arranged colonoscopic screening (Table 2). Among whites who participated in screening, 63.3% completed FIT and 36.7% completed colonoscopy. FIT rates were 59.2% for blacks, 62.7% for Hispanics, and 61.7% for Asians screened. (Table 2). Among those who utilized FIT, FIT was most often completed within 30 to 60 days of the initial mailing or not completed at all (Fig. 2).

For patients who underwent screening colonoscopy, 158 (25.9%) had non-advanced adenomas, 36 (5.9%) had advanced tubular adenomas and 6 (1.0%) had colon or rectal adenocarcinomas. The remaining colonoscopies had poor preparation (11 patients, 1.8%) and normal or other findings such as internal hemorrhoids or colitis (400 patients, 65.5%).

### Screening uptake by number of mailings

According to the intervention design, 1701 patients received one mailing while 3485 patients received two. The overall screening uptake at the end of 1 year was 67.8% for patients that received one letter and 16.9% for patients that received two letters. Among those that received one letter and completed screening, 73.2% were screened by FIT. Among those screened that received two letters, 46.2% were screened by FIT.

### Follow-up after positive FIT and diagnostic colonoscopy results

**Fig. 3:**
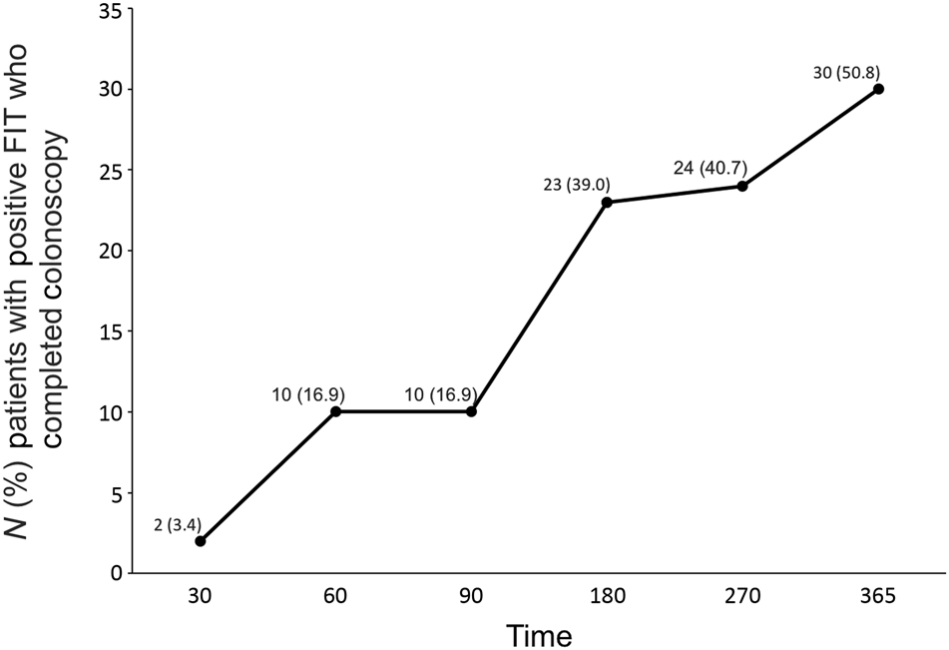
Number of patients with a positive fecal immunochemical test (FIT) who underwent diagnostic colonoscopy at 30 days, 60 days, 90 days, 180 days, 270 and 365 days. *N* (%)

There were 59 (5.5%) patients with positive FIT results. Of these, a total of 10 (16.9%), 23 (39.0%), and 30 (50.8%) completed a diagnostic colonoscopy within 90, 180, and 365 days, respectively (Fig. 3). At the end of the 1-year period, 29 (49.2%) participants had not completed a diagnostic colonoscopy after a positive FIT.

Results from diagnostic colonoscopies were as follows: 9 patients (30.0%) had non-advanced adenomas, 3 (10.0%) had advanced tubular adenomas, and 2 (6.7%) had a colon or rectal adenocarcinoma. Fifteen colonoscopies were normal (50.0%), and one case (3.3%) had poor preparation.

## Discussion

We implemented a multi-modal intervention to increase CRC screening rates among managed care patients in a large university-affiliated health system and observed a screening participation rate of 33.2% and a screening rate increase from 65.1% to 76.6%. The overall screening rate of 76.6% nears the NCCRT goal of 80% and is comparable to rates in other large integrated health systems with managed care populations such as Kaiser Permanente^8,24^. Additionally, our findings are consistent with a similar multi-level QI initiative that resulted in 23.6% screening uptake^22^. We hypothesize that our screening uptake rate was higher due to additional intervention components. Screening uptake rates in our HMO population were significantly higher in women compared to men and in Asians compared to whites. The FIT-positive rate of approximately 5% seen among our patients is comparable to other FIT mailing programs nationally^8,25^.

FIT screening occurred most often within the first 30 to 60 days of the initial mailing and declined thereafter, suggesting that patients’ willingness to participate in screening attenuates over time. We also noted that approximately 17% of patients required two mailings to comply with screening, supporting the use of serial outreach attempts. Colonoscopic screening most commonly occurred 90–180 days from the initial mailing, a delay likely explained by procedure scheduling logistics and wait-times.

Approximately half of the patients with positive FITs did not undergo diagnostic colonoscopy within 1 year, a finding consistent with prior studies that suggest a 40–60% colonoscopy follow-up rate after a positive FIT^26–31^. Of note, two positive FIT patients were deemed too ill to undergo colonoscopy and alternatively had CT colonography. Nonetheless, these findings suggest a need for further investigation into why rates of colonoscopic follow-up after positive FIT are low and for interventions to improve follow-up rates.

Several factors likely contributed to high screening uptake in our managed care cohort. Patients had a choice between two screening modalities, and data support that screening uptake is highest when patients are offered two or more screening modalities^32^. Our intervention also included several opportunities for contact with patients through repeated mailings and the use of a patient navigator. Prior studies demonstrated that direct mailing with follow-up reminders had a significant impact on increasing CRC screening rates^33,34^. Third, our intervention employed a patient navigator. Current peer-reviewed literature consistently highlights the benefits of patient navigation on CRC screening completion^11,35–37^ and colon cancer care^38,39^. Finally, our program enhanced communication between providers to facilitate CRC screening efforts, receipt of screening results and scheduling.

There are several strengths to our QI evaluation. Our multi-modal intervention centers on a multi-disciplinary approach to CRC screening; however, unlike other interventions reported in the literature, our findings highlight care coordination that includes GI specialists through streamlined management of abnormal results and coordinated scheduling efforts by our GI QI team. The intervention conserved PCPs in their roles as the primary liaison for patients, but aimed to offload PCPs by enhancing communication lines and decreasing intermediary steps. Second, we worked within a large, academic health care system with a diverse managed care patient population. All patients had access to insurance coverage with no out of pocket expense, which eliminated patient concerns about the costs. Additionally, strong financial incentives to stay within HMO insurance plans promoted care within our system and facilitated accurate collection of screening utilization data. Finally, we developed and engaged a multi-disciplinary QI team to provide oversight for appropriate screening follow-up and to optimize data capture and entry within the HM system.

Despite its strengths, our study is not without limitations. First, we included the managed care population from only one health care system, which may limit generalizability to other populations. However, this subgroup had a low screening rate despite adequate insurance coverage and an infrastructure to optimize screening utilization. Thus, we felt that this patient population was appropriate for our screening intervention. Second, many of the processes in our intervention relied on manual data entry and email communication for expedited procedure scheduling, both of which require resources that may limit the potential for intervention dissemination. Automation of such processes within current EMRs might limit the burden on health systems and increase intervention success. Third, we were unable to account for screening tests and colonoscopies performed outside of UCLA Health. However, patient enrollment in a managed care plan strongly dis-incentivizes out-of-network care. Our FIT mailings also prompted participants to provide information about screening completed at outside facilities, which minimizes the opportunity for missed screening. Additionally, while we were able to exclude patients with a documented history of CRC and/or total colectomy from the cohort, we were unable to exclude patients considered ineligible for screening due to high comorbidity. As a result, some participants may not have been appropriate for screening. Finally, because the study was completed as a large multi-component intervention, it is impossible to delineate the potential impact of individual components on CRC screening uptake rate. Many health systems may not be able to implement all components due to resource and cost limitations; nonetheless, this intervention was designed with the intention to synchronously address multiple barriers to screening.

In conclusion, our work supports the use of multi-modal interventions to increase CRC screening rates in an academic-affiliated health system managed care population. We also highlight the need for increased efforts to improve colonoscopic follow-up after positive FIT. Given that multiple patient, provider, and system barriers inhibit successful population health management, health systems should shift away from single-level interventions towards interventions with multiple components to address the multiple barriers to care delivery. With an increasing focus on quality of care, comprehensive approaches like these will help us achieve long-term control of CRC. Future QI efforts should develop and evaluate such interventions in other clinical settings, patient populations and payment models. Additionally, future work should determine and address barriers to diagnostic colonoscopy among those with positive FIT results.

## Study Highlights

### What is current knowledge?

- Colorectal cancer (CRC) screening rates are below the national goal of 80%.
- Single-component interventions aimed to improve screening uptake only modestly improve CRC screening rates.

### What is new here?

- Interventions that target multiple patient-, provider- and system-level barriers to CRC screening effectively increase screening uptake.
- Rates of diagnostic colonoscopy after positive fecal immunochemical test (FIT) are low and warrant further investigation.

### Translational impact

- Health systems should shift their focus from single-level to multi-level interventions when addressing barriers to CRC screening.
